# Optimizing the capture of neophobic rice field rats in lowland Asian rice ecosystems

**DOI:** 10.1002/ps.7043

**Published:** 2022-07-09

**Authors:** Renee P. Lorica, Alexander M. Stuart, Grant R. Singleton, Sudarmaji, Steven R. Belmain

**Affiliations:** ^1^ Sustainable Impact Platform International Rice Research Institute Los Baños Philippines; ^2^ Natural Resources Institute University of Greenwich Kent UK; ^3^ Pesticide Action Network UK, Brighthelm Centre Brighton UK; ^4^ Research Centre for Food Crops, Research Organization for Agriculture and Food National Research and Innovation Agency (BRIN); Cibinong Science Centre Bogor Indonesia; ^5^ Present address: ASEAN Centre for Biodiversity Los Baños Philippines

**Keywords:** rodent pests, live traps, ecologically‐based rodent management, rice, Asia

## Abstract

**BACKGROUND:**

Trapping is a key method for monitoring small mammals and is also one of a number of methods recommended under an ecologically‐based rodent management program to control rodent pest populations. Live‐traps are widely used globally for studying small mammal populations. In Asia where rodents are major pests of rice, single capture traps typically provide low trap success. We compared the trap success between two types of live‐traps in rice fields in Indonesia and the Philippines.

**RESULTS:**

Multiple‐capture traps (MCTs) in conjunction with a linear trap barrier were significantly more effective in catching rodent pest species than single‐capture traps (SCTs) in Indonesia and the Philippines. In Indonesia, MCTs captured more individuals with a mean (±SE) percent trap success rate of (15.54 ± 4.29) compared to SCTs (3.88 ± 1.58). In the Philippines, MCTs captured more species of rodents and had a significantly higher recapture rate (1.96 ± 0.79), than SCTs (0.58 ± 0.32).

**CONCLUSION:**

Multiple‐capture traps with a linear trap‐barrier were more effective for capturing *Rattus argentiventer* and *Rattus tanezumi* in rice field ecosystems compared to single‐capture traps. MCTs captured more species of rodent pests in the Philippines and recaptured more individuals of each species. These results indicate that rodent populations can be more effectively monitored and controlled by using a multi‐capture trap with barrier system than the use of single capture traps on their own. This is the first time these two trap types have been compared for use in rice ecosystems in Asia. © 2022 The Authors. *Pest Management Science* published by John Wiley & Sons Ltd on behalf of Society of Chemical Industry.

## Graphical abstract

Multiple‐capture traps with a linear trap barrier were more effective for capturing rodent pests in rice field ecosystems in Indonesia and the Philippines compared to single‐capture traps.

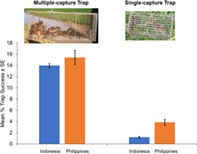

## 1 INTRODUCTION

Trapping is a key method for monitoring small mammals. There are many different trap types and techniques, and the choice depends on the purpose, the species and the habitat type.^1^ It has long been recognized that rate of success varies amongst different trap types.^2^, ^3^, ^4^ However, none of these studies have been conducted for rodents occurring in agricultural areas in Asia, save for the work of Motro, *et al*.,^5^ which compared 11 different trap types in Israel in areas planted to crops such as cereals, citrus, alfalfa, vegetables, and legumes. Notably, this study did not include rice cropping systems.

Rodents cause significant losses to rice both in Asia and Africa, which in turn has a major impact on the food security of smallholder farmers.^6^, ^7^, ^8^ Most of the world's rice is grown and consumed in Asia. Our study is in Southeast Asia where the two main rodent pest species of rice are *Rattus argentiventer* (Robinson & Kloss, 1916) and *Rattus tanezumi* (Temminck, 1844).^9^ Both these pest species have extraordinarily low recapture rates,^10^, ^11^, ^12^, ^13^, ^14^ which appears to suggest a high neophobia towards novel objects, as has been described in some *Rattus* species.^15^ There is little published information comparing the effectiveness of different live‐traps for population studies of these economically important pest species. This is particularly important to understand key factors that influence the population dynamics of pest rodent species, to develop management approaches based on our understanding of such dynamics, and to demonstrate effectiveness of control programs.^16^

Our study in the rice fields of Philippines and Indonesia is particularly focused on the environment of lowland rice growing areas that are typically found throughout Southeast Asia. These rice growing areas are typified by smallholder farming systems (most farms are <2 ha), often across very large flood plains where there are no other notable landscape features besides small village communities, the road network that connects them, and irrigation canals. As this is such an important Asian landscape where rodent pests cause considerable rice crop damage, we aimed to determine whether there is a difference between trap success, recapture rates, mortality rates and species diversity between single‐capture traps (SCTs), and multiple‐capture traps (MCTs) in combination with a linear trap barrier system (LTBS). Results of a large‐scale field study in West Java, Indonesia, indicated that *R. argentiventer* rarely entered live‐traps without a drift‐fence.^13^ Previous research in other parts of Indonesia also used multiple‐capture traps.^13^, ^17^, ^18^, ^19^ One research team used single‐capture traps to study the ecology of *R. argentiventer* in Indonesia where they caught *R. argentiventer* in SCTs set in a trapping grid of 11 × 18 stations in a 2 ha experimental field that was enclosed with rat‐proof fences.^19^, ^20^, ^21^ In Vietnam, single‐capture traps also have been used for population studies on *R. argentiventer*.^10^, ^22^, ^23^ However, comparative studies in using these different types of trap in the same environment are lacking. Although efficacy of each trap was our main priority, we also attempted to measure potential issues of humaneness by monitoring mortality of captured rodents. In addition to comparative trap efficacy and mortality, we also analyzed which trap type is a better investment than the other for research on the population ecology of rodent pest species and pest control purposes.

## 2 MATERIALS AND METHODS

### 2.1 Ethical approval

Live‐capture, handling, marking and euthanasia of rodents conformed to the 2016 Guidelines of the American Society of Mammalogists for the use of wild mammals in research and education^24^ and the AVMA Guidelines for the Euthanasia of Animals.^25^ Permits were secured from the Department of Environment and Natural Resources in the Philippines (R5‐74 and R4A‐WGP‐2017‐LAG‐003), and the Ministry of Research, Technology, and Higher Education in Indonesia (1169/FRP/E5/Dit.KI/V/2016 and 1192/FRP/E5/Dit.KI/VI/2017) prior to the conduct of the research.

### 2.2 Study sites

**Figure 1:**
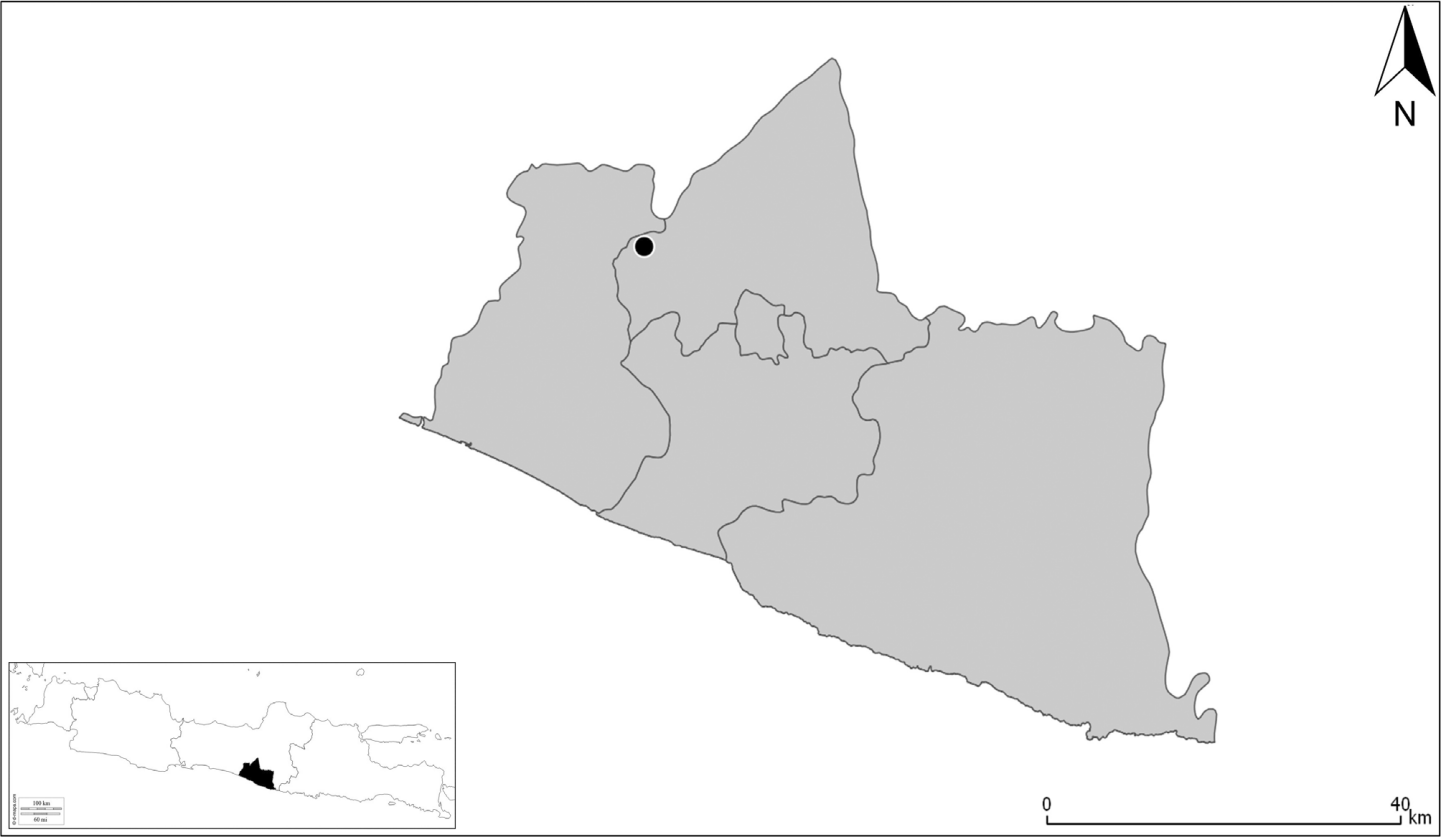
Location of the study area, Minggir, in the Special Region of Yogyakarta, in the Indonesian island of Java.

In Indonesia, the study was located within the Special Region of Yogyakarta in Minggir, Sleman Regency where farm field sites vary between 0.2–2.0 ha (S7° 43′ 42.38″, E110° 15′ 2.35″‐S7° 43′ 27.27″, E110° 15′ 10.17″; S7° 43′ 52.87″, E110° 15′ 13.33″‐S7° 43′ 31.79″, E110° 15′ 37.59″; Fig. 1). The farming system is dominated by lowland rice, forming a mosaic of villages with fruit and nut trees and rice fields over an area of approximately 150 km^2^ bordered by the Progo River to the North and West and the city of Yogyakarta to the east. Individual farmer fields vary in size from 0.2–1.0 ha with contiguous rice field areas of 100–200 ha between villages. All land is utilized, with no native habitat remaining, where field margins along rice bunds may contain local grass species, and where roadsides may contain occasional small shrubs or trees. Farmers were randomly selected from a much larger group who participated in a previous study on ecologically‐based rodent management (EBRM) from 2012–2014. The dominant rodent pest species in the area is *Rattus argentiventer*. Yogyakarta experiences type Am (tropical monsoon) climate according to the Köppen‐Geiger classification.^26^

**Figure 2:**
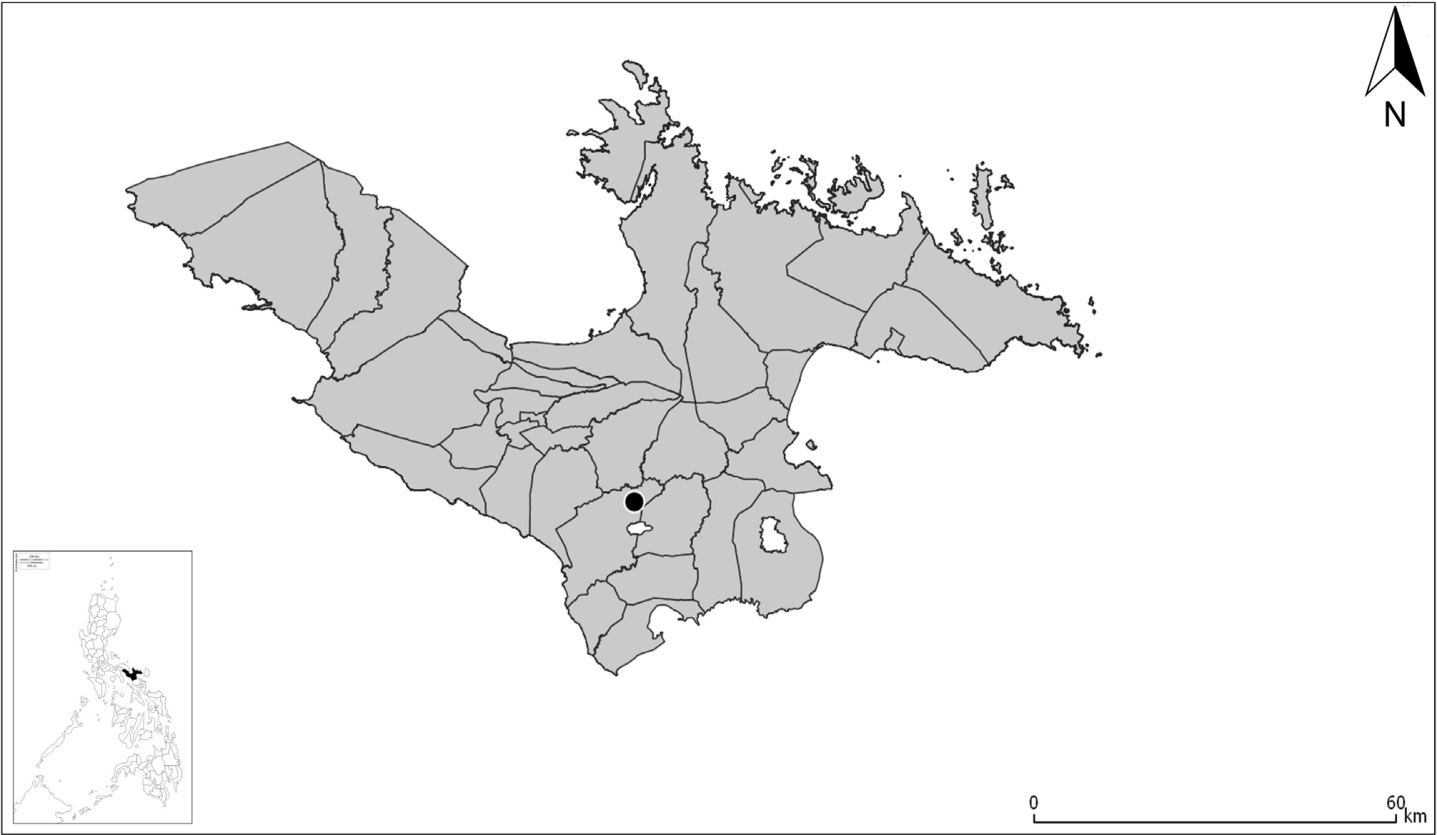
Location of the study site in Bula, Camarines Sur, Philippines.

In the Philippines, the study site was located in Bula, Camarines Sur, Bicol Region in Southern Luzon where farm field sites vary between 0.1–3.0 ha (N13° 30.404′ E123° 18.375′‐N13° 30.321′ E123° 17.614′; N13° 29.119′ E123° 18.052′‐N13° 29.319′ E123° 19.647′; Fig. 2). Farming systems are dominated by lowland rice over an area of 25 km^2^ bordered by the Pawili River to the North and West, Baao Lake to the South and the Mt. Iriga highlands to the Southeast. Farm sizes ranged from 0.1–3.0 ha with contiguous rice cropping areas of approximately 500 ha between villages. All land is utilized, with no native habitat remaining, where field margins along rice bunds may contain local grass species, and where roadsides may contain occasional small shrubs or trees. Large, mostly fruit and nut trees are primarily found in village areas. Farmers were randomly selected from a larger International Rice Research Institute (IRRI) project evaluating the adoption and impact of Alternate Wetting and Drying which encompassed the entire Rinconada Integrated Irrigation System (RIIS) in Camarines Sur, Bicol Region. The dominant rodent pest species in the area is *Rattus tanezumi*. This region has one of the highest rodent damage rates on rice in the Philippines (Singleton, unpublished data). The Köppen‐Geiger classifies Bula as Af (tropical rainforest) climate.^26^

### 2.3 Trapping design

**Figure 3:**
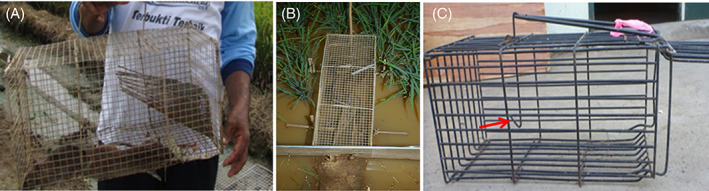
Multiple‐capture trap (A) and set against a drift‐fence (B). A single‐capture trap (C) set to spring, the red arrow indicates the hook on which the bait is suspended.

Two types of live‐cage traps commonly used in rice fields in Asia were compared. The multiple‐capture trap (MCT) is a wire mesh (1.27 cm) cage trap that has a cone at the opening tapering to the end of the wire facing inwards (240 mm long, 100 mm at the trap opening that tapers to 50 mm), which prevents captures from escaping through the entrance (Fig. 3(A)). MCTs can catch multiple animals at any time. It has a door on the other end fitted with a locking mechanism. Used in conjunction with a 0.6 m high × 100 m long drift‐fence (Fig. 3), it is called the Linear Trap Barrier System (LTBS).^27^ Each trap is set flush to the hole (12 × 12 cm‐sized holes, spaced 20 m apart, 10 cm from the ground) in the LTBS, alternating on either side of the fence, and suspended from the water by a mound of soil. The single‐capture trap (SCT) is a cage trap but with a hinged, sprung door triggered by the movement of a hook on which the bait is suspended (Fig. 3(C)). SCTs normally only trap a single animal.

Trapping of rodents was conducted over two rice cropping seasons: the dry season in 2016 and in 2017. Trapping was limited to the dry season as this was concurrent with an investigation the effect of intermittent irrigation on rodent pest ecology (see Lorica, *et al*.^28^). Traps were set for four nights during the following four stages of the rice crop: maximum tillering, panicle initiation/booting, flowering, and ripening. Replicate fields in each study site were similar in age of crop, area, and sufficiently proximate such that all replicates could be visited within 2 h, but far apart enough from each other that rodent populations in each replicate do not overlap. These criteria are crucial because all traps need to be checked with all rats processed and released before midday. Based on previous research on the home ranges of the main pest species per country,^29^, ^30^ each replicate had a minimum buffer radius of 170 m for *R. tanezumi* in the Philippines and 100 m for *R. argentiventer* in Indonesia.

In Indonesia, there were six replicate sites, which each included one LTBS with six MCTs (38 × 20.5 × 20.5 cm) as well as six single‐capture traps (SCT; 30 × 15 × 15 cm) spaced 10 m apart and placed at least 20 m away from the LTBS. Traps set to capture *R. argentiventer* were baited with boiled unmilled rice.^31^

In the Philippines, there were six replicate sites that included an LTBS with 15 multiple‐capture traps (33 × 20.3 × 20.3 cm) as well as 15 single‐capture traps. The MCTs were spaced 20 m apart while the SCTs (30 × 15 × 15 cm) were spaced 15 m apart in a line and placed at least 20 m away from the LTBS. Traps set to capture *R. tanezumi* were baited with fresh coconut and golden apple snail.^20^

Each trap was covered with vegetation to provide shelter from rain or sun. At completion of each trapping session, the traps were removed. Precipitation, crop stage and any rodent management done by the farmer during the trapping periods were recorded. Traps were set in the afternoon and checked early morning the following day for three consecutive nights per crop stage. Captured rats were ear marked with uniquely numbered ear tags, measured (head‐body length, foot length, ear length), sexed, weighed and reproductive condition recorded before the animal was released at site of capture. Ear tags were used as they are considered relatively harmless compared to other marking methods.^32^

### 2.4 Analyses

Analyses were conducted separately for data from Indonesia and the Philippines given the different farming practices and rodent pest species involved. Trap success was measured by the number of rodents captured divided by the trapping effort (total number of trap‐nights).^1^ A linear mixed model with maximum likelihood estimation was used to analyze the effect of trap type on number of captures. Repeated measures included in the model were year and crop stage. Fixed effects and their interactions were entered into a model that included year, crop stage, number of rats, and trap type. Each replicate site was considered a random effect. The penalized likelihood method using the lowest Akaike's Information Criteria (AIC) was used to determine best fit.^33^, ^34^

The number of recaptured individuals (recaptures), and the number of individuals found dead in the traps per replicate field site (mortality), regardless of year or species, were subjected to a paired samples T‐test to compare recaptures and mortality, respectively, of rodent pests between single‐ and multiple‐capture traps. A paired samples T‐test was used given that both trap types were sampling the same population of rodents. All statistical tests were performed using SPSS 24.0 for Windows.^35^

Cost per rat was calculated to determine the cost‐effectiveness of a trap type. Trap type costs included materials required to make the drift fence (plastic sheeting, bamboo, rat traps) and associated labor costs. Expenditure was calculated for each country in local currency and converted to US dollars for comparison.

## 3 RESULTS

### 3.1 Indonesia

#### 3.1.1 Trap success

**Figure 4:**
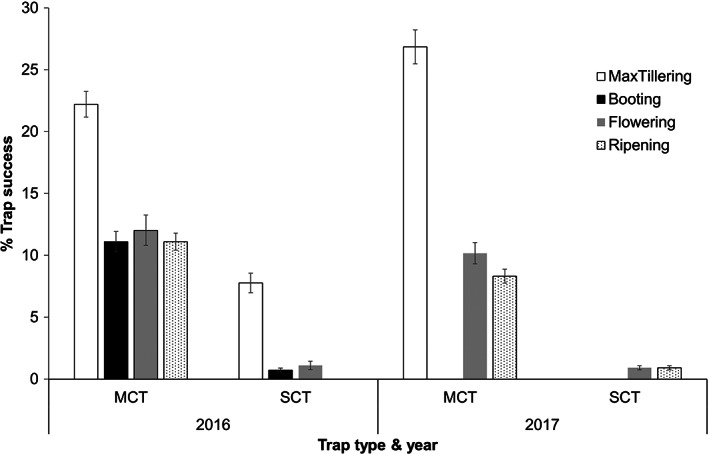
Precent trap success (number of rats caught per 100 trap‐nights; mean ± SE) for multiple capture trap (MCTs) and single capture traps (SCTs) across the growing stages of the rice crop in Yogyakarta, Indonesia, in 2016 and 2017.

In Indonesia, trap success was significantly affected by trap type (*F*_*1, 60.28*_ = 28.5*, P* < 0.001). Across both years, the trap success of MCTs was significantly higher than SCTs (Fig. 4). In 2016, the mean (±SE) percent trap success of MCTs and SCTs were 2.04 ± 0.45 and 0.46 ± 0.23, respectively. In 2017, the mean trap success of MCTS and SCTs were 2.72 ± 0.65 and 0.11 ± 0.08, respectively. The highest trap success was during the maximum tillering stage. However, year and crop stage effects were not significant (*P* > 0.05). Total trap nights for each trap type were 432 in 2016 and 324 in 2017. Due to inconsistent timing in transplanting between the replicates, trapping was missed for the booting stage in 2017. There were no recaptures of marked *R. argentiventer* individuals in either year. Only one other species was captured once by a single‐capture trap: the greater bandicoot rat (*Bandicota indica* Bechstein 1800). The mean mortality of *R. argentiventer* per replicate field site (±SE) did not differ (*t*_41_ = 1.704, *P* = 0.096) between MCTs (0.17 ± 0.06) and SCTs (0.048 ± 0.03).

#### 3.1.2 Cost

The materials and labor costs of establishing one LTBS with six multiple‐capture traps are detailed in Table 1. A commercial single‐capture trap could be readily purchased in Yogyakarta for IDR18,000 (Indonesian Rupiah to US Dollar, USD1.23 in 2016). However, multi‐capture traps are generally not commercially available and were made locally using wire mesh that was available in the local hardware stores. As multi‐capture traps use a passive capture design with no springs or triggers, they are easily made, so the MCTs were made by one of the local farmer leaders. The linear trap barrier was made by the rodent research group at the Institute of Rice Research in Sukamandi, Java. Cost of shipping the LTBS from Sukamandi to Yogyakarta was not included in the calculation. MCTs with LTBS captured a total of 98 *R. argentiventer* over two cropping seasons, effectively costing IDR25,867.35 per rat using the cost for the total number of LBTS constructed. On the other hand, a comparable number of SCTs without drift fencing captured a total of 13 *R. argentiventer* over two cropping seasons, effectively costing IDR49,815.15 per rat.

**Table 1:** Costs of establishment of one unit of Linear Trap Barrier System (LTBS) with 6 MCTs in Indonesia (USD1 = IDR14,580)

Item	Specification	IDR	USD
Drift‐fence	100 m × 60 cm; 6 trap‐holes	1755,00	120
Multiple‐capture traps	6 units/fence	480 000	33
Bamboo	1.5 m × 100 pcs	100 000	7
Labor	2 pax for 1 day	200 000	14
Total		2 535 000	174

### 3.2 Philippines

#### 3.2.1 Trap success

**Figure 5:**
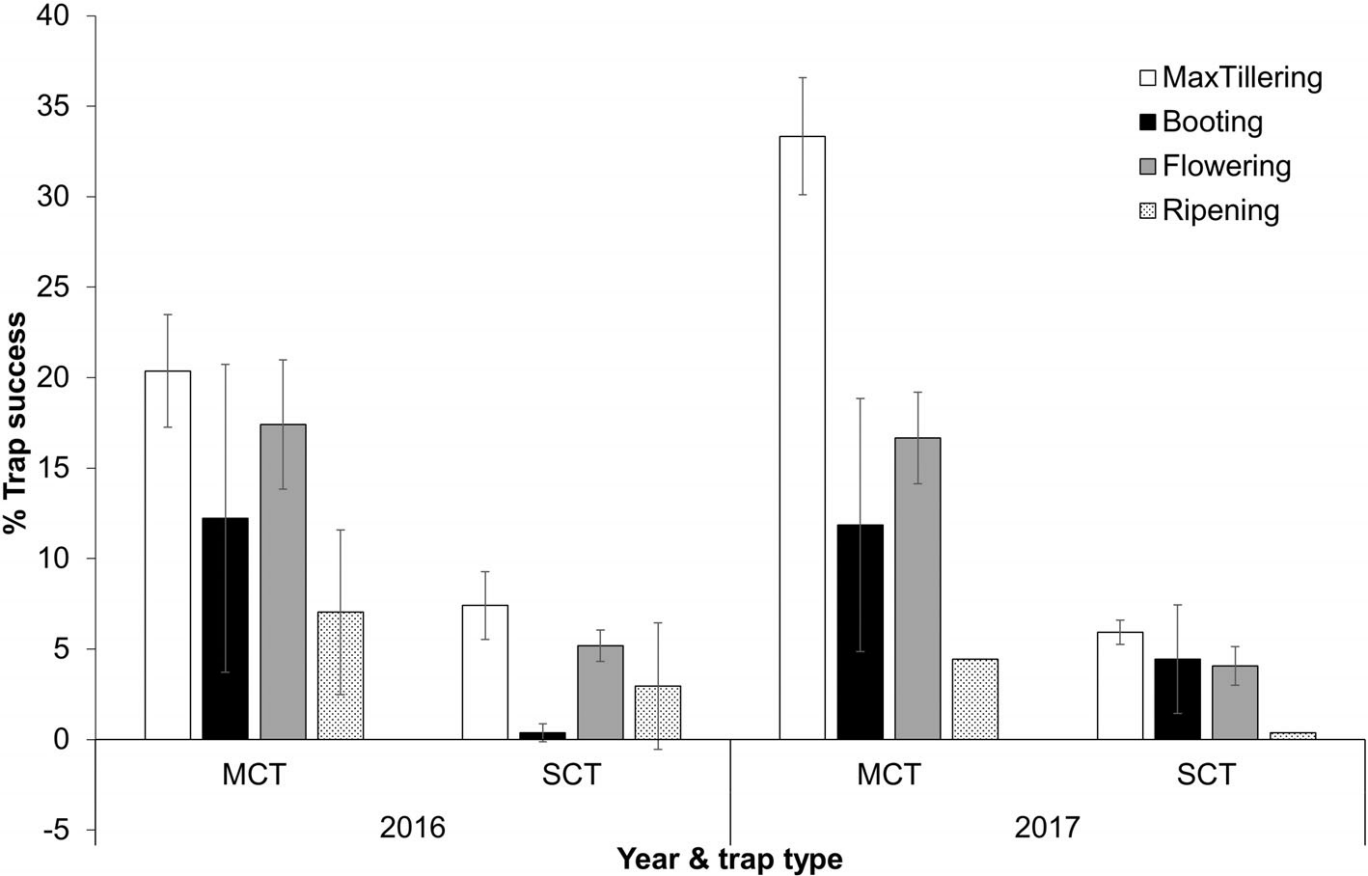
Percent trap success (number of rats caught per 100 trap‐nights; mean ± SE) for multiple capture trap (MCTs) and single capture traps (SCTs) across the growing stages of the rice crop in Bicol, Philippines in 2016 and 2017.

In the Philippines, trap success was significantly affected by trap type (*F*_*1, 16.70*_ = 64.313*, P* < 0.001). Across both years, the trap success of MCTs was significantly higher than SCTs (Fig. 5). In 2016, the mean trap success of MCTs and SCTs were 11.85 ± 2.01 and 3.31 ± 0.85, respectively. In 2017 the mean trap success of MCTS and SCTs were 12.79 ± 1.91 and 2.86 ± 0.62, respectively. The highest trap success was during the maximum tillering stage. However, year and crop stage effects were not significant (*P* > 0.05).

**Figure 6:**
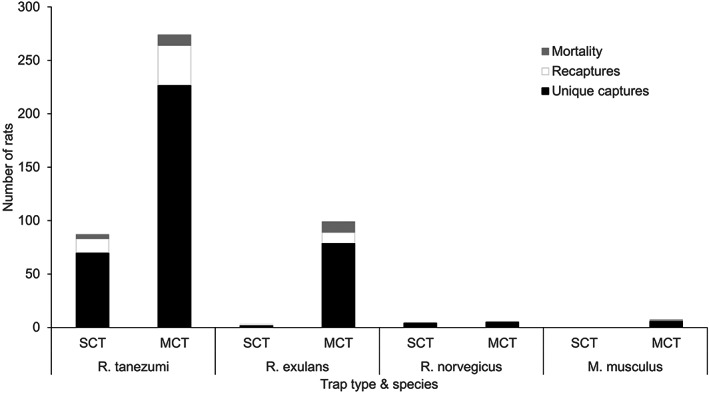
Proportion of unique captures, recaptures and mortality of the different species of rodent pest species in rice in Bicol, Philippines, for the 2 years of data gathering, allocated per trap type.

Figure 6 shows the proportion of unique captures, recaptures, and mortality for the different rodent pest species caught in the Philippines for the two types of traps. Three other rodent pest species were caught in Camarines Sur: the Polynesian rat (*Rattus exulans* Peale 1848), the Norway rat (*Rattus norvegicus* Berkenhout 1769), and the house mouse (*Mus musculus* Linneaus 1758), as well as the Asian house shrew (*Suncus murinus* Linnaeus, 1766). MCTs (15.54 ± 4.29) caught significantly more individuals of the different species (*t*_23_ = −3.682, *P* = 0.001) than SCTs (3.88 ± 1.58). SCTs did not catch any *M. musculus*. Recaptures for all species were also significantly higher (*t*_23_ = −2.2, *P* = 0.038) for MCTs (1.96 ± 0.79) than for SCTs (0.583 ± 0.32). However, mortality rates for each species in the traps were significantly higher (*t*_23_ = −2.9, *P* = 0.008) for MCTs (0.792 ± 0.23) than for SCTs (0.167 ± 0.10).

#### 3.2.2 Cost

The single‐capture traps were bought from local hardware stores for PhP374.78 (Philippine Peso to USD6.70) each. The costs of establishing one LTBS with six multiple‐capture traps are detailed in Table 2. Multiple‐capture traps were fabricated from galvanized iron welded wire mesh (1.27 cm mesh size). MCTs with LTBS captured a total of 385 rats over two cropping seasons, effectively costing PhP174.55 per rat. On the other hand, SCTs captured 94 rats over two cropping seasons, costing PhP23.92 per rat.

**Table 2:** Costs of establishing one unit of a Linear Trap Barrier System (LTBS) with 6 MCTs in the Philippines (1 USD = PhP56)

Item	Specification	PhP	USD
Drift‐fence	100 m × 60 cm; 6 trap‐holes	2900	54
Multiple‐capture traps	6 units/fence	7200	135
Bamboo	1.5 m × 100 pcs	500	9
Labor	2 pax for one day	600	12
Total		11 200	210

## 4 DISCUSSION

For both *R. tanezumi* and *R. argentiventer* in rice fields, the Linear Trap Barrier System (LTBS) with multiple‐capture traps was the more effective method of capture. This outcome aligns with other studies that indicate drift fences are important to capture neophobic species such as *R. argentiventer*.^13^, ^17^, ^18^, ^19^ Single capture traps have been successfully used, but often where study sites have been enclosed with rat‐proof fences.^19^, ^21^, ^31^ Studies on other species of rodents outside Asia have compared trappability between single‐ and multiple‐capture traps. In the wheat lands of north‐western Victoria, Australia, the trappability of house mice (*M. musculus*) in pitfall traps with a drift fence (another multiple‐capture method) was 30–40% during a high density period, *versus* 11–20% for Longworth traps (a single‐capture trap).^2^ However, at low to medium density periods, Longworth traps were more efficient in trapping mice (and keeping them alive) than the Ugglan multiple‐capture traps.^36^ Sherman traps, which are single‐capture traps, captured all known resident gerbils in a Negev Desert study site, whilst Ugglan traps did not capture a single animal.^37^ In the same paper, Ylönen *et al*. concluded that Ugglan traps were most efficient for trapping small mammals in boreal habitats with dense undercover, whilst Longworth traps are best for arid and open environments.

Trap success of rats may be influenced by trappability,^23^ prevailing environmental conditions, and human activities in agricultural fields, or a combination of these factors. Neophobic behavior to traps is well documented for some *Rattus* species (see Barnett^15^ for review). The lack of initial captures in our study further confirms that both species appear to have a degree of neophobia to novel objects such as live‐capture traps. The difficulty of catching *R. argentiventer* and extremely low recapture rates is well‐documented in different countries across their range in Southeast Asia^10^, ^23^, ^38^, ^39^, ^40^ and suggests that the species is likely trap‐shy. There was also no recaptures of *R. tanezumi* in live‐traps in a population study in Banaue, northern Philippines.^41^ This is why detailed capture‐mark‐recapture (CMR) analyses, whilst providing an absolute measure of abundance, was not used in our study given the extremely low recapture rates of both species. Compared to *R. argentiventer*, *R. tanezumi* entered live‐traps more readily, which is consistent with the general use of single‐capture live‐traps in population studies in the Philippines.^30^, ^41^ Within a species, trap‐shy and trap prone individuals have been documented for house mice (*Mus* musculus),^42^ bank voles (*Myodes glareolus*),^43^ grey squirrels (*Sciurus carolinensis*),^44^ and free‐ranging urban dogs (*Canis familiaris*).^45^ Trap‐shyness has not been previously documented for either *R. tanezumi* or *R. argentiventer* but may account for the relatively low trap success rates for both species when compared to a high level of rat damage to a rice crop, particularly when trapping in such a food‐rich environment.^13^, ^23^, ^46^

Another aspect that may determine trap‐shyness of a sub‐set of individuals in a population involves their dominance or subordination status. Dominant bank voles have been reported to have precedence over subordinate voles to baited traps.^47^ Moreover, dominant cotton rats did not avoid traps with conspecific odor whilst subordinate rats did.^48^ Social hierarchy has been rarely studied for either *R. tanezumi* or R. *argentiventer* so we cannot draw conclusions if this affected their trappability. Although one study of *R. argentiventer* in rice fields in West Java, Indonesia, concluded that low quality females (based on body mass and breeding condition) were more trap prone for a TBS.^21^ Differences in mean number of captures between multiple‐ and single‐capture traps may not be able to adequately reflect social hierarchy. More research is required on the effect of social status on the trap success of these two rodent species.

MCTs with the LTBS had a higher success in capturing more individuals of more species of small mammals compared to SCTs, at least for the Philippines, where it successfully caught all the species of small mammals found in the area. However, in Indonesia, the MCTs only caught *R. argentiventer*. The island of Java has four other rodent pest species inhabiting agricultural areas: the greater bandicoot *Bandicota indica*, the Norway rat (*R. norvegicus*), the black rat (*R. rattus*), and the Polynesian rat (*R. exulans*) but only one individual of one of these species was caught in the current study. In a 3‐year, monthly trapping study in an irrigated rice ecosystem in West Java from 1999–2002, 98.6% of rodent captures were *R. argentiventer*, indicating dominance of the species in that habitat type.^46^ *Rattus rattus diardii* and *B. indica* were only caught in rice fields adjacent to human habitation at the generative and ripening stages of rice in the same study. Indeed, the lone *B. indica* caught in this study was captured by an SCT in a rice field adjacent to a cemetery.

To maximize trapping effort for ecological studies on *R. tanezumi* and *R. argentiventer* in irrigated lowland rice fields, it is recommended that the Linear Trap Barrier System (with multiple‐capture trap) is used, which costs about USD200 in both Indonesia and the Philippines. However, it could be argued that single‐capture traps are lighter, easier to deploy in various types of environments, and more easily concealed to avoid being stolen (My Phung, pers. comm.) A single‐capture trap is cheaper than an LTBS on a per trap basis. However, those available in Yogyakarta were not effective in catching rats. In the Philippines, good‐quality single‐capture traps can be readily bought from a hardware store. In addition, there was a lower observed mortality rate in SCTs than MCTs, at least for the Philippines. However, when analyzing individual data for mortality, a disproportionate number were sexually immature males, regardless of the species (Lorica *et al*., unpublished data). Previous research on other species of small mammal suggest that younger animals may react more to the stress of capture,^49^ and adult male aggression towards conspecifics could also contribute to higher mortality in immature animals.^50^, ^51^, ^52^, ^53^ Providing better cover, increasing food availability, and increasing the frequency of checking the traps may help reduce trap mortality.^54^

For controlling rats in rice fields by farmers, the LTBS, whilst most effective, might be too costly and require high effort to maintain the fence.^55^ The drift‐fence was custom‐made for this project and the material used was of a quality that we were able to use the fence for several years in the field. Farmers may opt for a cheaper, though less durable material. The multiple‐capture trap used in Indonesia was made locally by a farmer, and tended to last only one season with daily use. Also, both kinds of traps had to be checked every morning and emptied of rats before re‐setting at dusk. This research supports the need for a LTBS to be managed at a community level and applied in strategic locations at key times to intercept rodents during a period of high dispersal.^56^ Alternatively, a modification of the LTBS, the Community Trap Barrier System (CTBS) has been shown to be even more effective to reduce rodent numbers and crop damage in irrigated rice fields both in Asia^13^ and Africa.^57^ A CTBS involves the establishment of a lure rice crop 2–3 weeks ahead of the surrounding irrigated rice field, and is enclosed by a 20–50 sq m TBS with multiple‐capture traps flushed with the TBS, and opening to the holes in the fence. A water‐filled moat surrounds the TBS, and elevated soil mounds serve as walkways, leading to the opening to the MCTs in the TBS.^58^ The CTBS provides a 200‐m radius halo of protection to approximately 16 ha of rice fields which was shown to be cost beneficial in Indonesia.^13^ In Vietnam, detailed modelling of rodent impacts in lowland rice systems using the APSIM‐Oryza rice model concluded that the CTBS approach was effective for the Vu3 crop in the Mekong Delta.^59^ The CTBS has also been effective in China (see Singleton, *et al*.^60^ for review).

## 5 CONCLUSION

Multiple‐capture traps with the Linear Trap Barrier System were more effective for capturing *R. argentiventer* and *R. tanezumi* in rice field ecosystems compared to single‐capture traps. MCTs captured more species of rodent pests in the Philippines and recaptured more individuals of each species. Regardless of trap type used, *R. argentiventer* is difficult to recapture apparently due to inherent trap‐shyness of the species. To effectively control rodent pest populations, the use of the Community Trap Barrier System is recommended.

## AUTHOR CONTRIBUTION

All authors were involved in the design of the study. RPL analyzed the data with AMS; she also implemented the field work with assistance from SDM. All authors contributed to the writing of the paper.

## CONFLICT OF INTEREST DECLARATION

The authors declare no conflict of interest.

## Data Availability

The data that support the findings of this study are available from the corresponding author upon reasonable request.
